# The production of Newcastle disease virus-like particles in *Nicotiana benthamiana* as potential vaccines

**DOI:** 10.3389/fpls.2023.1130910

**Published:** 2023-02-16

**Authors:** Tanja Smith, Martha M. O’Kennedy, Craig S. Ross, Nicola S. Lewis, Celia Abolnik

**Affiliations:** ^1^Department of Production Animal Studies, Faculty of Veterinary Science, University of Pretoria, Gauteng, Pretoria, South Africa; ^2^Next Generation Health, Council for Scientific and Industrial Research, Pretoria, South Africa; ^3^Avian Virology Department, Animal and Plant Health Agency (APHA), Woodham Lane, Addlestone, United Kingdom; ^4^Department of Pathobiology and Population Sciences, Royal Veterinary College, Hatfield, United Kingdom

**Keywords:** Newcastle disease virus (NDV), virus-like particles (VLPs), *Nicotiana benthamiana*, agroinfiltration, poultry vaccine, immunogenicity

## Abstract

Newcastle disease (ND) is a highly contagious viral respiratory and neurological disease that has a severe impact on poultry production worldwide. In the present study, an expression platform was established for the transient production in *N.bethamiana* of ND virus-like particles (VLPs) for use as vaccines against ND. The expression of the ND Fusion (F) and/or Hemagglutinin-neuraminidase (HN) proteins of a genotype VII.2 strain formed ND VLPs *in planta* as visualized under the transmission electron microscope, and HN-containing VLPs agglutinated chicken erythrocytes with hemagglutination (HA) titres of up to 13 log_2_.The immunogenicity of the partially-purified ND VLPs was confirmed in specific-pathogen-free White leghorn chickens. Birds receiving a single intramuscular immunization with 1024 HA units (10 log_2_) of the F/HN ND VLPs administered with 20% [v/v] Emulsigen^®^-P adjuvant, seroconverted after 14 days with F- and HN-specific antibodies at ELISA titres of 5705.17 and HI geometric mean titres (GMTs) of 6.2 log_2_, respectively. Furthermore, these ND-specific antibodies successfully inhibited viral replication *in vitro* of two antigenically closely-related ND virus isolates, with virus-neutralization test GMTs of 3.47 and 3.4, respectively. Plant-produced ND VLPs have great potential as antigen-matched vaccines for poultry and other avian species that are highly immunogenic, cost-effective, and facilitate prompt updating to ensure improved protection against emerging ND field viruses.

## Introduction

Newcastle disease (ND) is a viral avian disease that poses a significant problem to the poultry industry globally and is endemic in many countries (Ganar et al., 2014; Abolnik et al., 2017; Miller and Koch, 2020; World Organization for Animal Health (WOAH), 2022). The impact of ND on poultry production can be particularly severe in rural areas where backyard flocks are a source of income as well as a critical source of food (Martin, 1991; Awan et al., 1994; Alexander, 2000). Significant financial costs are incurred each year globally in the prevention of ND, in maintaining a ND-free status for export purposes, and/or to eradicate ND following an outbreak. Strict biosecurity measures in conjunction with good management and vaccination practices are imperative to prevent the occurrence and spread of this disease (Alexander, 2000; Dimitrov et al., 2016; Absalón et al., 2019; Miller and Koch, 2020). ND is notifiable to the World Organization for Animal Health (WOAH) and is caused by virulent strains of *Avian Orthoavulavirus 1* (AOAV-1) (formerly referred to as avian paramyxovirus serotype 1 (APMV-1) and avian avulavirus-1 (AAvV)), genus *Orthoavulavirus*, subfamily *Avulavirinae* (International Committee on Taxonomy of Viruses (ICTV), 2022). The ND virus has a non-segmented negative-sense RNA genome of ca. 15,192 bp that encodes six structural proteins: fusion protein (F), hemagglutinin-neuraminidase (HN) protein, Matrix protein (M), nucleoprotein (NP), phosphoprotein (P), and large polymerase (L) protein (Miller and Koch, 2020). The F and HN glycosylated proteins are anchored in and protrude from the surface of the viral envelope, which is derived from the host, with the M protein forming a monolayer on the inside of the viral envelope. The M protein is attached to the inner surface of the viral envelope and plays a role in the assembly of ND viral particles as well as viral budding. NP is the most abundant protein and protects the RNA genome from host nucleases by its encapsidation (“nucleocapsid core”). The NP-RNA nucleocapsid core forms a complex with the P and L proteins to form a ribonucleoprotein (RNP) complex, which is involved in the transcription and replication processes of the NDV. Two additional non-functional proteins, V and W, are generated as a result of polymerase slippage of the *P* gene during transcription (Steward et al., 1993). The V protein could play a role in viral replication and evading the host’s innate immune system (Chu et al., 2018; Sun et al., 2019), while the function of the W protein remains unclear at this time. Following viral attachment and fusion to the host cell membranes, which is mediated by the F and HN proteins, respectively, the viral nucleocapsid complex is released into the host cell. After viral replication in the cytoplasm, viral particles are assembled and progenies bud from the cell surface (Miller and Koch, 2020). Following infection (or vaccination), the F and HN glycoproteins are the primary targets for neutralizing antibodies (Arora et al., 2010; Kim et al., 2013).

ND has a wide host range, with more than 200 species of wild birds and poultry species susceptible to infection (Alexander, 2000; Miller and Koch, 2020; World Organization for Animal Health (WOAH), 2022). Viral transmission occurs through shedding *via* the oropharyngeal and cloacal routes, whereafter susceptible birds can be infected either by inhaling or ingesting the virus. Clinical signs of ND vary greatly depending on the strain and host species, and can range from a drop in feed and water intake and/or egg production in layer hens to a mortality rate of up to 100% in unvaccinated birds (Miller and Koch, 2020; World Organization for Animal Health (WOAH), 2022). The onset of symptoms usually occurs between two and fifteen days following natural exposure to NDV, although it may take up to four weeks in some cases (Miller and Koch, 2020). Depending on the clinical signs in specific-pathogen-free (SPF) chickens following experimental inoculation, four pathotypes of disease are used to describe the virulence of ND viral strains. In descending order of virulence, the pathotypes are as follows: 1) velogenic (high morbidity/mortality), 2) mesogenic (high morbidity/low mortality), 3) lentogenic (low morbidity/mortality) and 4) asymptomatic enteric (Miller and Koch, 2020). All NDV isolates are classified into a single serotype, but ND isolates are genetically and antigenically diverse and are continually undergoing evolutionary changes (Dimitrov et al., 2016; Dimitrov et al., 2019; Miller and Koch, 2020). ND isolates are phylogenetically grouped into two classes based on their full genome sequences: class I includes one genotype and isolates are predominantly of low virulence, while class II includes 20 genotypes and isolates are of low and high virulence (Dimitrov et al., 2016; Dimitrov et al., 2019). The host tropism and geographic distribution of the respective genotypes differ, with some exclusively infecting certain avian species in a limited geographic area (Dimitrov et al., 2016; Dimitrov et al., 2019). With the incidence of ND on the rise and the considerable antigenic drift of ND isolates that occurred over time (Dimitrov et al., 2017; Miller and Koch, 2020), the control of ND is critical.

Vaccination against ND is commonly practised around the world and plays a critical role in disease control. Vaccination offers protection against clinical disease and morbidity and can reduce the amount of virus shed upon exposure to a field isolate, consequently limiting the spread to new susceptible hosts. As lentogenic (and asymptomatic enteric) viral strains cause asymptomatic infection in SPF chickens, they are commonly used to develop attenuated-live and inactivated egg-produced ND vaccines for commercial poultry (Collett and Smith, 2020). Live vaccines are suitable for mass application and elicit both cellular and humoral responses, although the immunity can be short-lived (Dimitrov et al., 2017; Collett and Smith, 2020; Nurzijah et al., 2022; World Organization for Animal Health (WOAH), 2022). Inactivated vaccines are considered to be safer than live vaccines and induce good humoral responses but require direct immunization (intramuscularly or subcutaneously), as well as formulation with an adjuvant and/or multiple vaccine doses to enhance the immunogenicity. Egg-based vaccine production is a well-established and cost-effective technology, although both live and inactivated vaccine production involve the handling of infectious viral material, which poses biosafety concerns and necessitates biocontainment facilities. Furthermore, due to the genetic variation between the F and/or HN sequences of lentogenic vaccine strains and contemporary ND isolates, the capacity of these live and inactivated vaccines to confer protection and effectively reduce viral shedding in vaccinated birds is likely reduced (Miller et al., 2013; Dimitrov et al., 2017). Recently, recombinant viral vector vaccines expressing the ND HN and/or F proteins have been approved for use. Recombinant viral vector vaccines effectively stimulate both humoral and cellular immune responses, like live vaccines, but with superior safety, a shorter production time, and Differentiating Infected from Vaccinated Animals (DIVA)-potential. The production of these recombinant viral vector vaccines using the established low-cost egg-based production platform has been extensively investigated, although their production in cell culture systems is also possible, albeit at a higher production cost. For poultry vaccines, low manufacturing costs are imperative to ensure production margins are maintained (Dimitrov et al., 2017; Fulber and Kamen, 2022; Nurzijah et al., 2022; World Organization for Animal Health (WOAH), 2022).

As another alternative to traditional live and inactivated vaccines, the use of virus-like particles (VLPs) as vaccines have become increasingly popular in recent years. VLPs are self-assembling protein shells that display target epitopes of the native virus in a dense arrangement on its surface without any viral genetic material at their core, making them highly immunogenic and non-infectious (biocontainment facilities are not required) (Noad and Roy, 2003). VLPs have been produced using various prokaryotic and eukaryotic expression systems and their suitability as vaccine antigens to confer protect against various viral pathogens is well documented (Pushko et al., 2013). The omission of viral structural proteins also facilitates DIVA-compliance following vaccination of animals with VLP vaccines using appropriate serological tests (Park et al., 2014). ND VLPs have been produced using cell culture-based systems by co-expressing NDV F, HN and/or M (Pantua et al., 2006; Qian et al., 2017; Xu et al., 2019) and ND VLPs as vaccines proved to be highly effective against viral challenge in chickens in comparison to a commercial live ND vaccine (Xu et al., 2019). The use of plants to transiently produce VLP vaccines creates a scalable and cost-effective production platform that does not entail the use of viral material, endotoxins, or animal-derived reagents, and has an unprecedented emergency response capacity (D’Aoust et al., 2010; Kolitilin et al., 2014; Walwyn et al., 2015; Nandi et al., 2016; Morris et al., 2017; Tusé et al., 2020). Agroinfiltration refers to the process whereby a laboratory strain of a soil pathogen (*Agrobacterium tumefaciens)* is used to transport the gene of interest (incorporated into a selected plant expression vector) from the extracellular spaces in plant leaf tissue to the nucleus of the plant cell for transcription and subsequently expression of the heterologous protein, typically within 7 days (Sainsbury and Lomonossoff, 2008). As such, it is considered to be the most suited platform in the face of a pandemic or bioterrorism threat (Morris et al., 2017; Sainsbury, 2020; Tusé et al., 2020), as well as for infectious disease that entail rapidly evolving viruses and/or viruses with a great genetic and antigenic diversity (such as ND) for which the generation of antigen-matched vaccines might be required to match circulating viral strain a specific geographic region and/or a specific species. In addition to the cost-effective scalability and short production time of transient plant-based expression, this platform also facilitates *N*-glycosylation of the glycoprotein, which is important for the immunogenicity as well as the stability of VLPs (Kim et al., 2014). In this study the production of VLPs displaying NDV F and/or HN glycoproteins using *Agrobacterium*-mediated transient expression in *Nicotiana benthamiana* was investigated, the minimal combination of proteins required to obtain intact, functional ND VLPs was established, various parameters were optimized to maximize protein yield, and the immunogenicity of the VLPs as potential vaccines in chickens, as well as *in vivo* ND virus neutralization ability of the elicited antibodies, was established.

## Experimental procedures

### Synthetic gene design and plant expression vector construction

Synthetic codon-optimised genes were designed based on the unmodified HN and M protein sequences of NDV isolate turkey/South Africa/N2057/2013 (GenBank accession number: KR815908). For the *F* gene, the native signal peptide sequence was replaced with the *Mus musculus* monoclonal antibody heavy chain variable region signal peptide sequence (O’Hara et al., 2012). For each of the synthetic genes, *Age*I and *Xho*I restriction enzyme recognition sites were incorporated at the 5’- and 3’-terminals, respectively, for cloning into the pEAQ-HT vector (Sainsbury and Lomonossoff, 2008). The three genes (*F*, *HN* and *M*) were codon-optimized and synthesized by GeneArt™ (Thermo Fisher Scientific, Germany).

The three synthetic genes were each cloned into pEAQ-HT (Fast-Link™ DNA Ligation kit, Epicentre; Diagnostech, South Africa), transformed into competent DH10B *Escherichia coli* cells using electroporation, and kanamycin-resistant clones were screened *via* PCR with pEAQ-HT-specific primers, as previously described (Smith et al., 2020). PCR positive DH10B clones were inoculated into Luria Bertani (LB) media (1% tryptone [w/v], 0.5% yeast extract [w/v], 1% NaCl [w/v]) and incubated overnight (37°C, 200 rpm) for plasmid DNA isolation (Zyppy™ Plasmid Miniprep kit; Zymo Research). Plasmid DNA was submitted to Inqaba Biotech (Pty) Ltd. (Pretoria) for Sanger sequencing. Sequence-verified pEAQ-HT+F, pEAQ-HT+HN, and pEAQ-HT+M plasmids were each subsequently transformed into *Agrobacterium tumefaciens AGL*-1 competent cells using electroporation, as previously described (Smith et al., 2020). *Agrobacterium* strain *AGL*-1 was obtained from the American type culture collection (ATCC^®^BAA-101TM, *Rhizobium radiobacter*). Antibiotic-resistant *AGL*-1 clones were selected and verified to contain the respective genes of interest *via* PCR as described before, using pEAQ-HT-specific primers.

### Agroinfiltration of *N. benthamiana* with transformed *AGL*-1 clones

The validated transformed *AGL*-1 clones were infiltrated into the leaves of five-to-eight-week-old *N. benthamiana* plants (80-120 mm in height) modified to reduce plant-specific glycosylation patterns (Strasser et al., 2008) by hand (Shamloul et al., 2014). *A. tumefaciens* cultures containing pEAQ-HT+F, pEAQ-HT+HN, and pEAQ-HT+M, respectively, were sub-cultured and incubated overnight at 28°C in LB containing 30 μg/ml rifampicin, 50 μg/ml carbenicillin, and 50 μg/ml kanamycin, pelleted by centrifugation at 7,000xg for 7 minutes, and resuspended in infiltration buffer (10 mM 2-N-morpholino-ethanesulfonic acid (MES), 20 mM MgSO4, 200 μM Acetosyringone, pH 5.6). The respective infiltration mixes were diluted to obtain a final optical density at 600 nm of 1 to 2, whereafter different combination of plasmids were prepared. The inocula were incubated at room temperature for an hour and introduced into the leaves by hand, using a syringe without a needle.

At 4, 5 or 6 days post infiltration (dpi), the leaves were harvested and homogenised in two volumes of extraction buffer supplemented with sodium metabisulfite (0.04% [w/v]) (Landry et al., 2010) and proteinase inhibitor cocktail (P2714; Sigma-Aldrich) using a Matstone DO9001 Juicer. Two buffers previously used for the extraction of VLPs from infiltrated plant leaves were compared to maximize yield: 1 x phosphate buffered saline (PBS) buffer (4.3 mM sodium phosphate (NaHPO4), 1.4 mM monopotassium phosphate (KH2PO4), 2.7 mM potassium chloride (KCl), and 127 mM NaCl, pH 7.4) and Bicine buffer (50 mM Bicine, 20 mM NaCl, 0.1% [w/v] NLS sodium salt, pH 8.4) (Thuenemann et al., 2013; Smith et al., 2020). The slurry was clarified through a double layer of cheese cloth and centrifuged (7,000 x g, 7 minutes, 4°C), whereafter the clarified plant extract was partially purified using differential ultracentrifugation. The supernatant was loaded on top of a double sucrose (20%/70% sucrose; 1.5 ml of each sucrose layer) or triple sucrose (20%/40%/70% sucrose; layers 20% and 70% were 1.5 ml each while layer 40% was 1 ml) density gradient in a 13.2 ml Thinwall Ultra-Clear™ centrifuge tube (Beckman Coulter). After ultracentrifugation (32,000 x g, 2 hours, 10°C; Beckman Coulter Ultra-centrifuge Optima L90K), 0.5 ml fractions were collected from the bottom of the centrifuge tube.

### Biochemical analysis and confirmation of identity

The partially-purified proteins were analysed using sodium dodecylsulphate-polyacrylamide gel electrophoresis (SDS-PAGE) and immunoblotting. For SDS-PAGE analysis, protein was separated on 12% TGX Stain-Free™ FastCast™ acrylamide gel (Bio-Rad) under reducing conditions and stained with Coomassie G-250. For confirmation of identity, SDS-PAGE protein bands corresponding to the expected fragment size of each of the target proteins (F ±56 kDa; HN: ± 62 kDa; M: ± 40 kDa) were excised, in-gel trypsin digested (Shevchenko et al., 2007), and analysed by liquid chromatography-mass spectrometry (LC-MS/MS)-based peptide sequencing as previously reported (Chhiba-Govindjee et al., 2018). For immunoblotting, protein was separated on a 12% TGX Stain-Free™ FastCast™ acrylamide gel (Bio-Rad) under reducing conditions and transferred using a Trans-blot Turbo Midi 0.2 µm PVDF transfer pack and a Trans-blot Turbo blotter (Bio-Rad), according to manufacturer’s recommendation. Blocking was performed overnight in 1 x PBS containing Tween 20 (0.1% [v/v]) and 3% [w/v] Bovine Serum Albumin Fraction V (BSA). Plant-produced NDV F and NDV HN partially-purified *via* differential centrifugation were detected using standard NDV hyper immune antiserum prepared from a La Sota/46 strain ND virus (1:600; Deltamune Pty (Ltd), Pretoria, South Africa) in combination with goat anti-chicken IgY horseradish peroxidase (HRP) conjugated antibody (1:1,500; Novex Life Technologies, A16054; Thermo Fisher Scientific™), as well as a polyclonal NDV-F1- horseradish peroxidase (HRP) antibody (1:2000; CliniSciences, France). The proteins on the NDV antiserum blots were visualized using Chemiluminescence detection (Clarity™ Western ECL Blotting Substrate; Bio-Rad), while the proteins on the Anti-F1 NDV blots were visualised using Colorimetric detection (3,3’,5,5’-Tetramethylbenzidine (TMB) liquid substrate system for membranes, Thermo Fisher Scientific) using the ChemiDoc™ MP Imaging System (Bio-Rad).

The formation of ND VLPs was visually confirmed using negative-staining transmission electron microscopy (TEM). Carbon-coated copper grids (mesh size 200) were floated on 15 µl of the partially-purified protein sample for five minutes, whereafter the grid was washed five times by floating on 5 µl of sterile water. The particles were subsequently negatively-stained with uranyl acetate for 30 seconds. A Philips CM10, 80 kV transmission electron microscope or a JEOL 1400 Flash microscope was used for imaging (University of Pretoria, Electron Microscopy Unit). Two-fold serial dilutions of the partially-purified ND VLPs were tested for the ability to agglutinate chicken erythrocytes and the HA titre was calculated as the reciprocal of the dilution wherein complete hemagglutination occurred (World Organization for Animal Health (WOAH), 2022).

### Immunogenicity in chickens

#### Experimental animals

Six-week-old SPF White Leghorn Chickens (*Gallus gallus*) (n=10) purchased from Avi-Farms (Pty) Ltd, Pretoria were identified individually with numbered neck tags. The birds were housed together on sawdust with perches provided in an isolation room in the Biosafety Level 3 facility at the University of Pretoria’s Veterinary Faculty. Water and feed (Nova Feeds, South Africa) were provided *ad libitum*. All study procedures were approved by the Research and Animal Ethics Committees of the University of Pretoria (UP REC013-21; UP Animal Ethics Committee REC188-21), the CSIR (REC330/2020; REC384/2021) and the Department of Agriculture, Land Reform, and Rural Development (Section 20 permit no. 12/11/1/1/12).

#### Vaccine preparation

Six days after infiltration, 18g of infiltrated leaves was harvested and homogenized in PBS as previously described. ND VLPs were partially purified using density gradient ultracentrifugation (70%/20% sucrose), whereafter the NDV F/HN VLPs in sucrose fractions 2 to 4 were pooled, dialyzed in 1 x PBS overnight using a 3500 MWCO Slide-A-Lyzer protein dialysis cassette (Thermo Fisher Scientific), and trehalose (15% [w/v]) was added as stabilising agent. Partially-purified NDV F/HN VLPs were tested by hemagglutination assay (see serological testing) and stored at 4°C until use. The vaccine dose of 25 µl partially-purified ND F/HN VLPs was calculated to correspond to an HA titre of 10 log_2_ or 1024 HAU. On the day of vaccination, the partially purified plant extract containing the ND VLPs (25 µl) were either diluted in 1 x PBS to a final volume of 250 µl (non-adjuvanted treatment group; supplemented with 1% [v/v] enrofloxacin (Baytril^®^ 100, Bayer Animal Health, South Africa), or diluted in 1 x PBS and mixed with a commercial oil-in-water adjuvant Emulsigen^®^-P (MVP adjuvants^®^, Phibro Animal Health Corporation, United States; 20% [v/v]) plus 1% [v/v] enrofloxacin (Baytril^®^ 100, Bayer Animal Health, South Africa) to a final volume of 250 µl (adjuvanted treatment group). The Emulsigen-P commercial oil-in water adjuvant was selected due its effectiveness in a previous study conducted by the UP/CSIR research group involving the testing of a plant-produced VLP vaccine in poultry (Abolnik et al., 2022*)*.

#### Experimental design

Prior to vaccination (day 0 of the study), 1 ml blood was collected from the wing vein of each chicken to confirm that the birds were not previously exposed to NDV. The first treatment group (referred to as the non-adjuvanted treatment group) (n=5) was vaccinated intramuscularly in the breast muscle with 250 µl of the ND VLP vaccine formulated without an adjuvant, while the second treatment group (referred to as the adjuvanted treatment group) (n=5) was vaccinated intramuscularly in the breast muscle with 250 µl of the ND VLP vaccine formulated with Emulsigen^®^-P as adjuvant. Two weeks after immunization (14 days post vaccination (14 dpv)), blood was collected from all 10 chickens as above to determine the F- and HN- specific antibody responses and the chickens were humanely euthanized. Although prime-boost vaccination is typically applied to generate maximum antibody responses for challenge studies, a single dose was administered in this study as the aim was to assess whether the plant-produced ND F/HN VLPs were immunogenic in chickens.

#### Serological testing

Sera were separated by centrifugation at 3000 x g for 10 minutes at 4°C and transferred to sterile Eppendorf tubes. NDV F-specific antibodies were detected using the BioChek Newcastle Disease Virus Antibody Test kit (NDV-F ELISA), according to manufacturer’s instructions. The average sample to positive control ratio (S/P) of four replicates were determined, which was used to calculate the average antibody titre. A S/P ratio ≥ 0.300 is considered as positive, while and antibody titre ≥ 993 is considered as positive. NDV HN-specific antibodies were detected using Hemagglutination inhibition (HI) testing with standard NDV hyperimmune chicken NDV (La Sota) antiserum (Deltamune) by the diagnostic serology laboratory of the University’s Department of Veterinary Tropical Diseases, according to the standard procedures (World Organization for Animal Health (WOAH), 2022). HI titres ≥ 4 log2 were considered as positive.

Antisera collected from the adjuvanted treatment group (n=5 birds vaccinated with ND VLPs + Emulsigen^®^-P) on day 14 post vaccination were sent to the Animal and Plant Health Agency (APHA)-Weybridge, United Kingdom, for HI analysis and virus neutralisation testing. Six homologous and heterologous NDV isolates from different genotypes were selected from the APHA repository (**Supplementary Figure 1**), with the amino acid homology being the basis for selection. Although the ND VLP homologous virus (ND isolate turkey/South Africa/N2057/2013) was not available in the APHA repository, an isolate (St. Helena) with more than 99% sequence homology was included. HI testing against each of the six NDV isolates were performed in triplicate, with sera collected from chickens vaccinated with a La Sota-based ND vaccine available for use at APHA included as a positive control. Viruses were used at 4 haemagglutinating units (4 HAU) and HI results interpreted according to standard procedures (World Organization for Animal Health (WOAH), 2022). Virus neutralisation tests (VNT) were formed against each of the six antigens (in triplicate), with serum obtained from chickens vaccinated with a La Sota ND vaccine again serving as positive control. Sera were diluted two-fold in Dulbecco’s minimal essential medium (DMEM) supplemented with penicillin (100 U/ml) and streptomycin (100 μg/ml), 100 TCID_50_ of selected APMV-1 were added and the samples incubated at 37°C for one hour. Each of the mixtures was subsequently added to DF1 chicken fibroblast cells (ATCC; CRL-12203) for one hour and incubated at 37°C, before the virus/antibody mix was removed by aspiration and replaced with DMEM supplemented with 2.5% [v/v] embryonate fowls’ eggs (EFE) allantoic fluid, penicillin (100 U/ml) and streptomycin (100 μg/ml). Cells were incubated for five days at 37°C, whereafter the cytopathic effect was recorded.

## Results

### Transient expression of Newcastle disease VLPs in *N. benthamiana* plants

ND VLPs containing the equivalent F, HN and/or M viral proteins of isolate turkey/South Africa/N2057/2013, a virulent sub-genotype VII.2 strain (Abolnik et al., 2017; Dimitrov et al., 2019), were transiently produced in *N. benthamiana* plants lacking plant-specific N-glycosylation using agroinfiltration. Different combinations of ND proteins were co-expressed to determine the minimal protein required for VLP assembly in *N. benthamiana* plants. *Agrobacterium* inoculum containing the 1) pEAQ-HT+F, 2) pEAQ-HT+F and pEAQ-HT+M, 3) pEAQ-HT+HN, 4) pEAQ-HT+HN and pEAQ-HT+M, or 5) pEAQ-HT+F, pEAQ-HT+HN and pEAQ-HT+M recombinant plasmids were infiltrated into the leaves of five-to-eight-week plants. On the optimal day of harvesting (i.e., six days after infiltration, data not shown), the leaves were collected and homogenized in two volumes of extraction buffer supplemented with sodium metabisulfite. The plant extract was clarified using cheese cloth and centrifugation, whereafter the target proteins were partially-purified using differential ultracentrifugation.

**Figure 1:**
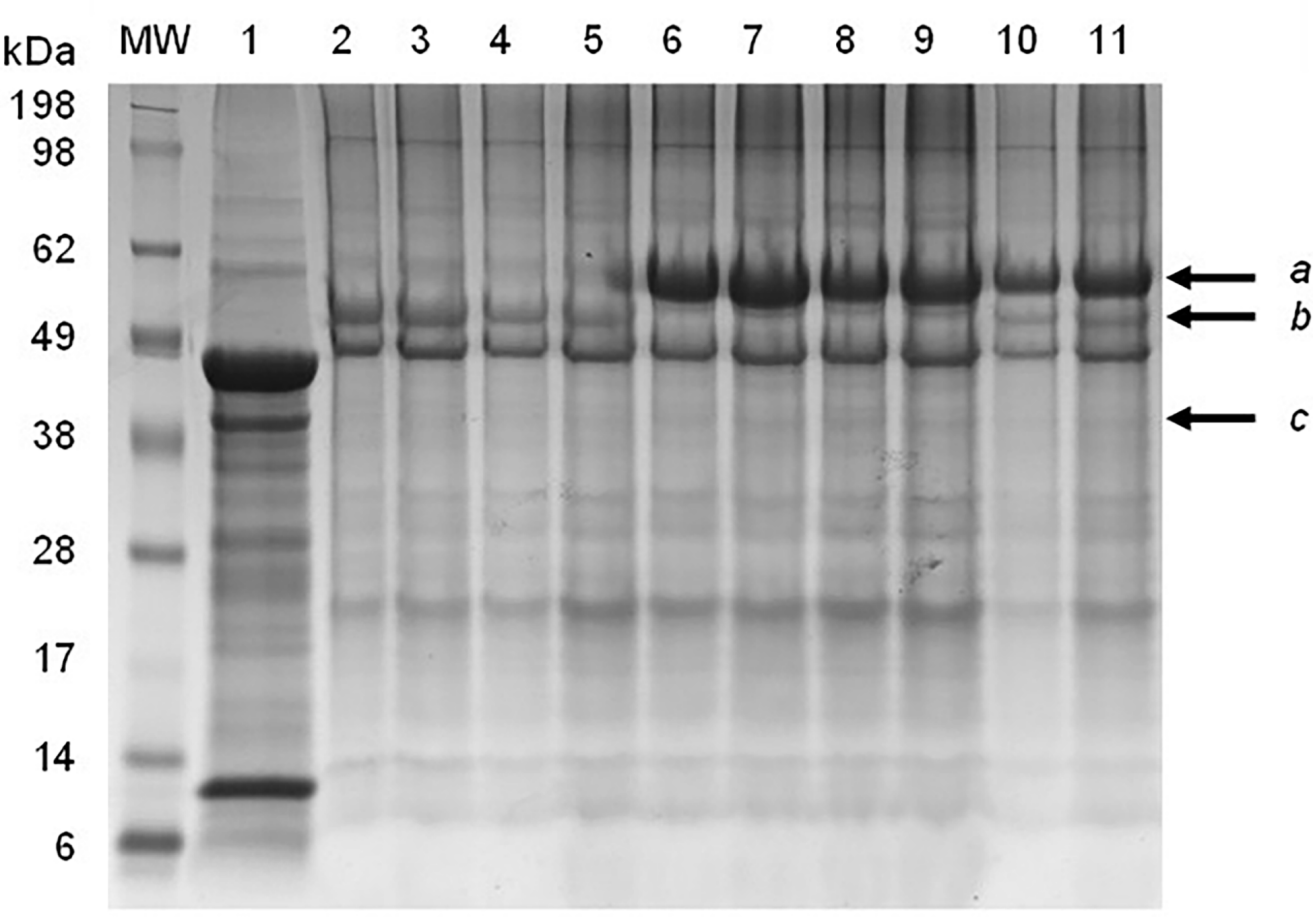
SDS-PAGE electrophoresis of partially-purified plant-produced ND VLPs obtained with different combinations of co-expressed Fusion (F), hemagglutinin-neuraminidase (HN) and/or Matrix (M) proteins. Lane 1: negative control – plant-expressed pEAQ-HT; lanes 2&3: F only; lanes 4&5: F + M; lanes 6&7: HN only; lanes 8&9: HN + M; lanes 10&11: F + HN + M. Arrows indicate the position of the (a) HN (approximately 62 kDa), (b) F (approximately 56 kDa) and (c) M (approximately 40 kD) proteins.

**Figure 2:**
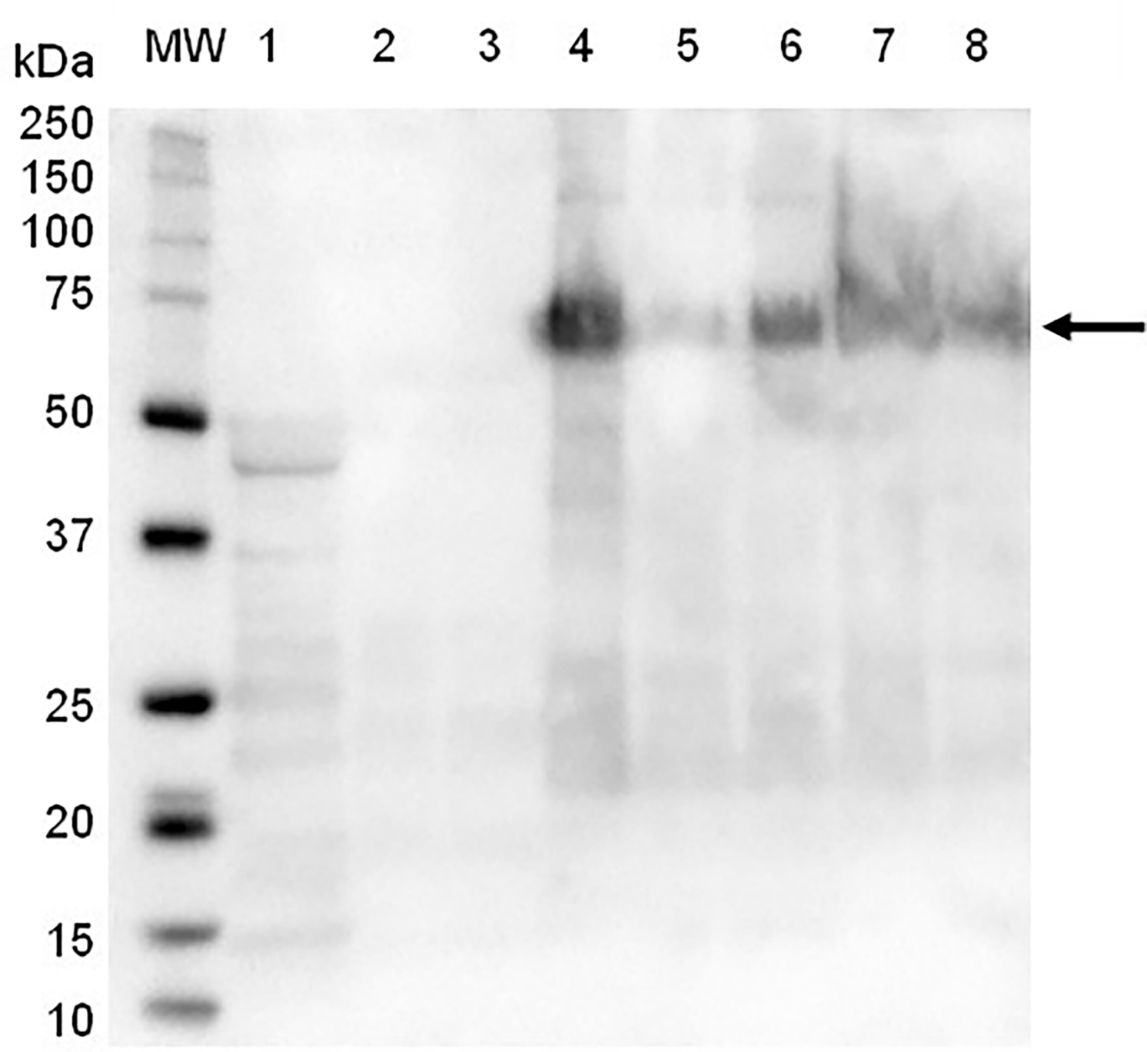
Immunoblot of partially-purified plant-produce ND VLPs obtained with the co-expression of NDV Fusion (F), hemagglutinin-neuraminidase (HN) and/or Matrix (M) proteins, using NDV antiserum and goat-anti-chicken IgY-HRP as secondary antibody. Lane 1: negative control – plant-expressed pEAQ-HT; lane 2: F only; lane 3: F + M; lane 4: HN only; lane 7: HN + M; lanes 5, 6 and 8: F + HN + M . Lane MW: The WesternC protein molecular weight marker was used. The arrow indicates the positions of the HN protein (approximately 62 kDa).

**Figure 3:**
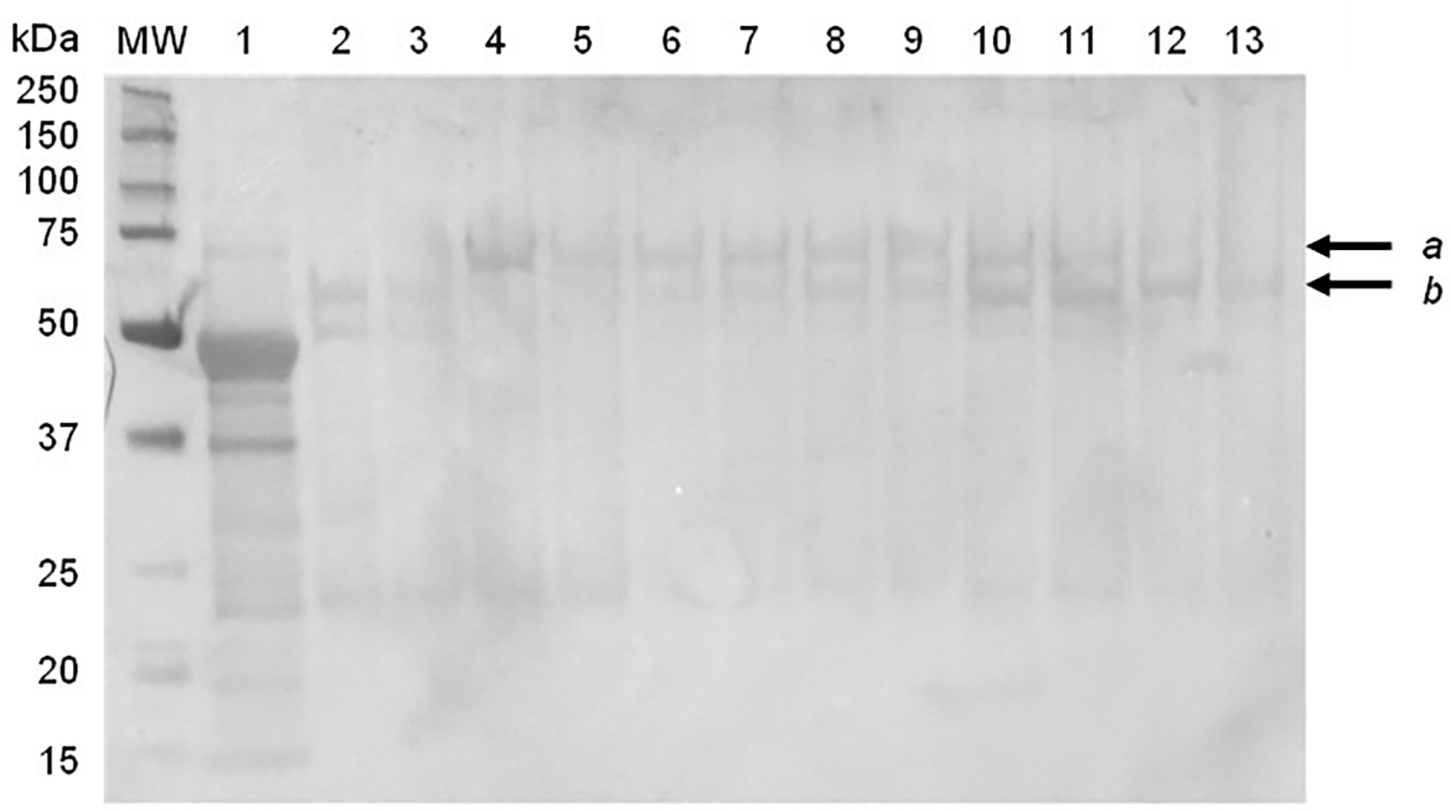
Immunoblot of partially purified plant-produce ND VLPs obtained with the co-expression of NDV Fusion (F), hemagglutinin-neuraminidase (HN) and/or Matrix (M) proteins, using NDV F1-specific antibody. Lane 1: negative control – plant-expressed pEAQ-HT; lane 2: F alone; lane 3: F + M; lane 4: HN alone; lane 5: HN + M; lanes 6-8: F + HN + M; lanes 9-13: F + HN. Lane MW: The WesternC protein molecular weight marker was used. Arrows indicate the position of the (a) HN (approximately 62 kDa) and (b) F (approximately 56 kDa) proteins.

**Figure 4:**
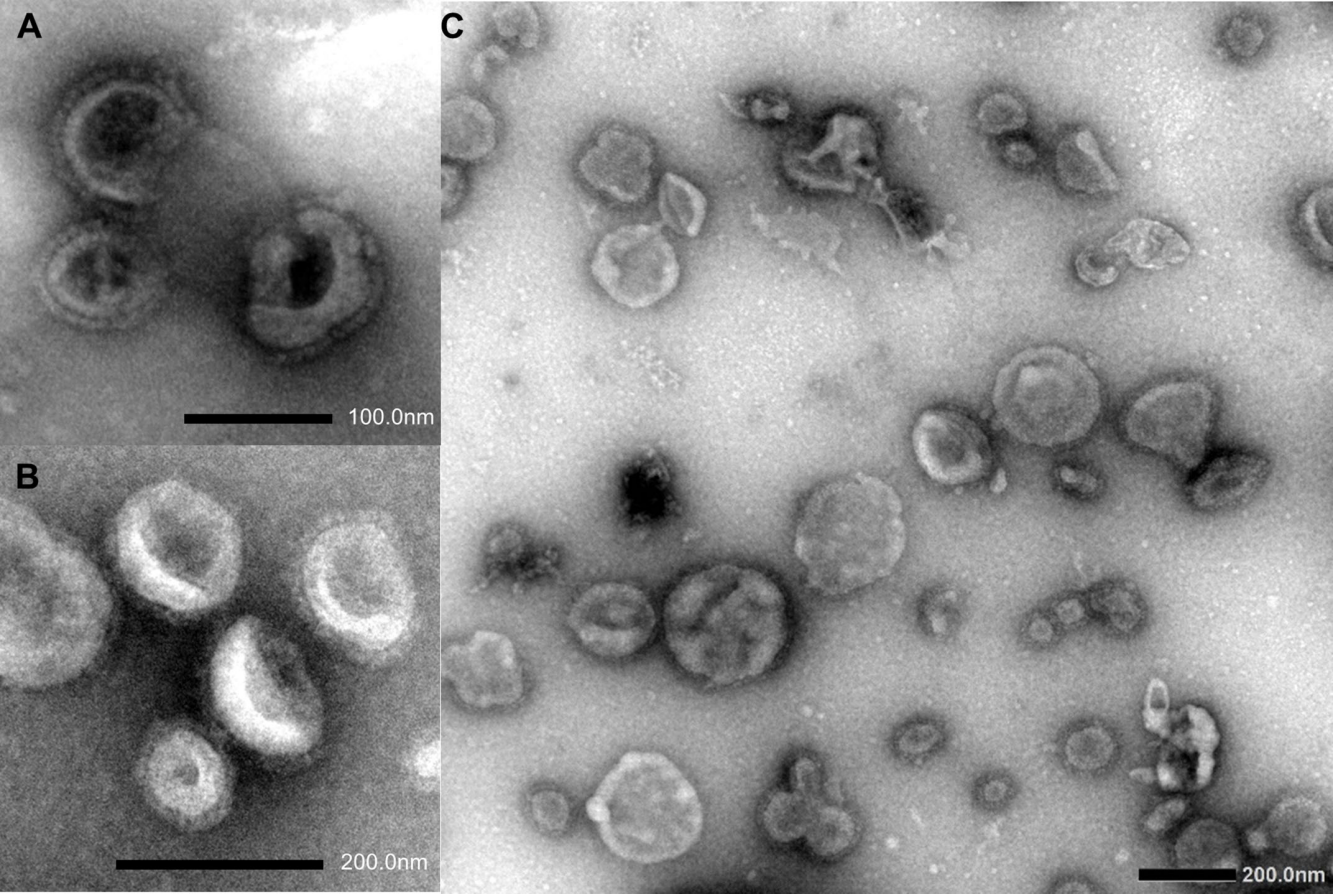
Negatively-stained transmission electron microscopy images of plant-produced Newcastle disease VLPs consisting of Fusion (F) **(A)**, hemagglutinin-neuraminidase (HN) **(B)**, or F + HN glycoproteins **(C)**.

SDS-PAGE and immunoblot analysis of partially-purified plant extract indicated prominent bands of approximately 56 kDa and 62 kDa, which correspond to the expected molecular weight of the synthetic F and HN viral proteins, respectively (**Figure 1**). The M protein has a molecular weight of approximately 40 kDa, but a distinct protein band at the expected position was not clearly identifiable using SDS-PAGE (**Figure 1**). The presence of NDV F and HN protein were confirmed *via* immunoblotting: NDV antiserum in combination with a goat anti-chicken IgY-HRP secondary antibody detected the HN protein (**Figure 2**), while an anti-NDV F1-HRP antibody detected the F protein (and surprisingly the HN protein as well) (**Figure 3**). LC-MS/MS-based peptide sequencing of the 56 kDa, 62 kDa and 40 kDa protein bands on the SDS-PAGE confirmed the plant-based production of the NDV F (81.4% coverage, 48 unique peptides; 95% confidence threshold), HN (72.7% coverage, 51 unique peptides; 95% confidence threshold), and M (21.4% coverage, 9 unique peptides; 95% confidence threshold) viral proteins (**Supplementary Figure 2**). Particles that resemble native ND viral particles were successfully produced in the apoplast when F (**Figure 4A**) and HN (**Figure 4B**) were expressed on their own, as well as with the co-expression of F and HN (**Figure 4C**). The VLPs were pleiomorphic but roughly spherical and typically ranged from 80 to 200 nm in diameter (**Figure 4**), although particles between 60 and 400 nm in diameter were observed. This is in line with native NDV viral particles, which replicate and assemble in the cytoplasm and bud from the cell surface, and are pleiomorphic. ND virions can be round with a diameter of 100 - 500 nm, or filamentous with a diameter of approximately 100 nm and variable lengths (Miller and Koch, 2020). To ensure maximum yield, two extraction buffers previously used for the purification of VLPs transiently produced in *N. benthamiana* using the pEAQ-HT vector were compared (Thuenemann et al., 2013; Smith et al., 2020). PBS buffer was found to be as effective as a Bicine-based buffer for the extraction of ND VLPs, and the addition of N-Lauroylsarcosine sodium salt (NLS) [a component of the Bicine buffer used by Thuenemann and colleagues (2013)], to the PBS buffer did not enhance the yield (data not shown). In contrast to reports of ND VLP expression in insect cells (Pantua et al., 2006; Qian et al., 2017; Xu et al., 2019), the co-expression of NDV M with F and/or HN is not required for ND VLP assembly in plants, nor did it result in a noticeable enhancement in protein yield (**Figures 1**, **2**). Functionality of the plant-produced HN-based ND VLPs were confirmed using hemagglutination assay (HA), as the HN protein is responsible for the agglutination of erythrocytes, a characteristic of ND viruses (Miller and Koch, 2020). HA titres ranging from 9 log_2_ (1: 512) to 13 log_2_ (1:8192) were consistently obtained for the HN-based ND VLPs.

### Assessing the immunogenicity of the plant-produced ND VLPs in chickens

The immunogenicity of the partially- purified ND F/HN VLPs was assessed in 6-week-old specific pathogen-free (SPF) White Leghorn chickens (n=10). To verify that the chickens were not previously exposed to NDV and as a baseline, the birds were bled prior to vaccination and the sera assessed *via* hemagglutination inhibition (HI) and BioChek NDV-F ELISA testing. All 10 birds tested negative with HIs and BioChek ELISAs (**Table 1**), confirming the absence of ND-specific antibodies prior to vaccination. The chickens were vaccinated once with either the partially-purified plant-produced ND VLPs on their own or the partially-purified plant-produced ND VLPs formulated with Emulsigen^®^-P, and no signs of adverse reactions at the injection site were observed. At 14 days post vaccination (dpv) the chickens were bled and the sera tested again using HI and the BioChek ELISA. All the birds in the adjuvanted treatment group tested positive for NDV HN-specific antibodies (with HI titres ranging from 5 log_2_ to 8 log_2_) and NDV F-specific antibodies (mean antibody titre of 5705.17) (**Table 1**). In contrast, all of the birds in the non-adjuvanted group tested negative for NDV antibodies at 14 dpv (**Table 1**), indicating that the plant-produced ND VLPs require administration with an appropriate adjuvant to elicit a humoral response.

**Table 1:** Serology test results for specific-pathogen-free chickens prior to vaccination and at 14 days post vaccination with positive values indicated in bold.

Treatment group	Bird number	Day 0			Day 14		
		F protein ELISA S/P ratio		HI Log_2_ titre Antibody Titre	F protein ELISA Average S/P ratio		HI Log_2_ titre Antibody Titre
ND VLPs without adjuvant	10331	0,07	239,88	2	0,10± 0.07	347,27	2
	10332	0,02	53,70	1	-0,02± 0.02	0	2
	10333	0,04	134,90	1	0,14± 0.04	467,46	1
	10334	0,03	107,15	1	0,03± 0.14	101,14	2
	10335	0,00	0	1	0,03± 0.03	108,24	2
	GMT	0.03± 0.03	107.13± 90.45	1.2± 0.45	0.06± 0.06	204.82± 194.49	1.8± 0.45
ND VLPs + Emulsigen^®^- P	10336	-0,01	0	1	**0,92±** **0.12**	**3047,34**	**6**
	10337	-0,01	0	1	**1,91±** **0.10**	**6325,96**	**8**
	10338	0,01	40.74	1	**0,45±** **0.09**	**1481,69**	**5**
	10339	0,05	173.78	1	**3,54±** **0.26**	**11731,97**	**6**
	10340	-0,01	0	1	**1,79±** **0.38**	**5938,89**	**6**
	GMT	0.01± 0.03	42.90± 75.26	1	**1.72±** **1.19**	**5705.17 ± ** **3926.33**	**6.2±** **1.10**

GMT, Geometric mean titre; HI, hemagglutination inhibition; S/P, sample to positive control.

The sera collected 14 dpv from the five chickens in the adjuvanted treatment group were subsequently analysed to assess the neutralization capacity of the elicited NDV-F and -HN antibodies against six NDV isolates (**Tables 2**, **3**). The highest HI geometric mean titres (GMTs) of 8.53 ± 1.51 log_2_ were obtained against the genotype XIII.2 AV264/18 antigen (93.14% amino acid sequence homology with the VLP vaccine strain), followed by the AV1260/17 (genotype XIV; 93.86% amino acid sequence homology with the VLP vaccine strain; HI GMT 6.53 ± 1.06 log_2_), St. Helena (genotype VII.2; 99.5% sequence homology with the VLP vaccine strain; HI GMT 5.67 ± 1.18 log_2_), and Macedonia/2020 (genotype VII.2; 91.8% sequence homology with the VLP vaccine strain) (HI GMT 5.33 ± 0.98 log_2_) antigens (**Tables 2**, **3**). A high HI titre did not necessarily equate to virus neutralization, and only two of these antigens, AV1260/17 (VNT GMT 3.47 ± 0.83 log_2_) and St. Helena (VNT GMT 3.4 ± 1.06 log_2_) yielded positive VNT titres for all five test serum samples, whereas virus neutralization against AV263/18 (high HI titre) was poor.

**Table 2:** Newcastle disease virus isolates selected for hemagglutination inhibition and virus neutralization testing.

NDV isolate	GenBank Accession number	Genotype	Amino acid sequence homology with F of the ND VLP vaccine strain^†^
Herts/33	AY741404	IV	88.8%
AV593/15 (PPMV-1/pigeon/UK/015874/15)	n/a	VI	87.0%
AV264/18 (Sweden) (APMV-1/Sweden/Chicken/007597/2017)	n/a	XIII.2	93.14%
AV1260/17 (Nigeria) (APMV-1/Nigeria/047852/2016)	n/a	XIV	93.86%
Macedonia/2020 (AOAV-1/chicken/Macedonia/1400-1/2020)	MT424733	VII.2	91.8%
St. Helena (APMV-1/St Helena/Chicken/027301/2014)	n/a	VII.2	99.5%

†The plant-produced VLP’s homologous virus is NDV isolate turkey/South Africa/N2057/2013).

n/a, not available, sequences are not available in the public domain.

**Table 3:** Serology results for hemagglutination inhibition and virus neutralization tests against six Newcastle disease viruses, with positive titres indicated in bold.

Serum	NDV isolate														
	Herts/33		AV593/15		AV264/18 (Sweden)			AV1260/17 (Nigeria)			Macedonia/2020			St. Helena	
	HI Log_2_ titre	VNT Log_2_ titre	HI Log_2_ titre	VNT Log_2_ titre	HI Log_2_ titre	VNT Log_2_ titre		HI Log_2_ titre	VNT Log_2_ titre		HI Log_2_ titre	VNT Log_2_ titre		HI Log_2_ titre	VNT Log_2_ titre
10336	**4**	**2**	2	1	**10**	0		**7**	**2**		**6**	**2**		**6**	**5**
	**4**	**3**	2	1	**10**	0		**7**	**5**		**6**	**3**		**6**	**4**
	**4**	**3**	2	1	**10**	0		**7**	**4**		**6**	**2**		**6**	**4**
10337	**6**	**5**	**4**	**3**	**9**	1		**8**	**4**		**7**	1		**7**	**4**
	**6**	**4**	**4**	**3**	**10**	1		**8**	**4**		**7**	**2**		**7**	**3**
	**6**	**3**	**4**	**3**	**10**	0		**8**	**4**		**6**	1		**8**	**4**
10338	2	0	0	0	**6**	0		**5**	**3**		**4**	**2**		**4**	**2**
	2	0	0	0	**6**	0		**5**	**3**		**4**	1		**4**	**2**
	2	0	0	0	**6**	0		**5**	**3**		**4**	1		**4**	**3**
10339	**4**	1	1	**2**	**9**	1		**6**	**4**		**5**	1		**5**	**5**
	**4**	**2**	1	**2**	**9**	1		**6**	**4**		**5**	1		**5**	**4**
	**4**	1	1	1	**9**	1		**6**	**4**		**5**	1		**5**	**4**
10340	**4**	0	1	0	**8**	0		**7**	**3**		**5**	**3**		**6**	**2**
	**4**	0	1	0	**8**	0		**7**	**2**		**5**	**3**		**6**	**3**
	**4**	0	1	0	**8**	0		**6**	**3**		**5**	**2**		**6**	**2**
**GMT**	**4±** **1.31**	1.6± 1.68	1.6± 1.40	1.13± 1.19	**8.53±** **1.51**	0.33± 1.49		**6.53±** **1.06**	**3.47±** **0.83**		**5.33±** **0.98**	1.73± 0.80		**5.67±** **1.18**	**3.4±** **1.06**
La Sota –control	**10**	**8**	**8**	**7**	**11**	**7**		**11**	**9**		**10**	**8**		**9**	**7**
	**10**	**8**	**8**	**7**	**11**	**7**		**11**	**9**		**10**	**8**		**9**	**7**
	**10**	**8**	**8**	**5**	**10**	**7**		**11**	**9**		**10**	**9**		**9**	**7**
**GMT**	**10±** **0**	**8±** **0**	**8±** **0**	**6.33±** **1.15**	**10.67±** **0.58**	**7±** **0**		**11±** **0**	**9±** **0**		**10±** **0**	**8.33±** **0.58**		**9±** **0**	**7±** **0**

GMT, Geometric mean titre; HI, hemagglutination inhibition; VNT, virus neutralization test.

## Discussion

The use of transient plant-based expression of VLP vaccines against economically important infectious diseases like ND holds enormous potential for animal health (Kolitilin et al., 2014; Sainsbury, 2020; Nurzijah et al., 2022). Insect cell-produced VLP vaccines against ND have been reported (Pantua et al., 2006; Qian et al., 2017; Xu et al., 2019), but successful VLP production using transient plant-based expression have not been documented before now (Nurzijah et al., 2022). The multiple advantages of VLPs produced by transient plant-based expression over other systems led to the ND VLPs investigated in the present study.

Abundant Newcastle disease VLPs displaying at least one ND glycoprotein were produced in *N. benthamiana* leaves using agroinfiltration. Contrary to previous reports involving ND VLPs produced in cell culture expression systems (Pantua et al., 2006; Qian et al., 2017; Xu et al., 2019), the NDV matrix protein was not required for ND VLP formation in *N. benthamiana* leaves and co-expression of M with the F and/or HN proteins did not lead to a detectable increase in VLP yield. According to Nurzijah et al. (2022), relative low yields and signs of necrosis from 5 dpi were obtained with the transient expression of NDV HN using the pEAQ-HT plant expression vector. In the present study signs of necrosis was observed from 4 days post infiltration with the expression of the NDV F protein, although this was mitigated by reducing the density (OD_600_) of the infiltration mixture to 1 which allowed harvesting of infiltrated leaves at the optimal day (6 dpi). Incorporating both the F and HN into the VLP as a vaccine is desirable, since protection increases when both HN and F are used together compared to the protection induced by vaccines that express F or HN alone (World Organization for Animal Health (WOAH), 2014).

The use of VLPs as vaccine antigens has become popular due to their effectiveness as immunogens, with both humoral and cellular immune responses being elicited (Bright et al., 2007; Quan et al., 2007; Landry et al., 2010). The VLPs are taken up by dendritic cells (DCs), the most potent antigen presenting cells (APCs), for processing and presentation by major histocompatibility complex (MHC) class II or I molecules for activation of CD4+ T cells (Th2 immune response) or CD8+ T cells (Th1 immune response), respectively. An adjuvant might, however, be used to enhance the immunogenicity of the vaccine, to reduce the number of immunizations (i.e., single vaccination *vs*. primer-boost regime) and/or to reduce the concentration of VLPs used per vaccine dose. With inactivated ND vaccines an adjuvant (commonly mineral oil-based) is employed to enhance the immunogenicity (Collett and Smith, 2020). Previously, ND HN and/or F antigens produced using either transient or stable expression in plants showed variable immunogenicity in animal studies (Nurzijah et al., 2022). In the present study the partially-purified plant-produced ND F/HN VLPs were formulated without and with a commercial adjuvant and tested for immunogenicity in SPF chickens. The addition of an adjuvant was necessary to elicit strong humoral responses, with the adjuvanted ND F/HN VLPs yielding good levels of HN-specific antibodies 14 days post vaccination. Furthermore, these antibodies demonstrated neutralising effects against two NDV isolates belonging to the homologous genotype VII and genotype XIV.

The capacity to differentiate between infected and vaccinated animals (DIVA) is of great importance for disease surveillance, diagnostic and/or export purposes (ND-free status). Due to the absence of internal viral proteins, VLP vaccines have DIVA-potential with the use of a combination of appropriate serological tests (Liu et al., 2013). In the present study, ND HN- and F-specific antibodies were detected using routine tests (HIs and an anti-NDV F commercial ELISAs, respectively) following vaccination with the plant-produced NDV F/HN VLPs. By making use of another commercial ELISA kit that targets antibodies against an internal ND protein, for example the ID Screen^®^ ELISA (ID.Vet, France) that detects nucleoprotein (NP)-specific antibodies, DIVA could be attained by confirming the absence of NP-specific antibodies in the sera that tested positive for F- and/or HN-specific antibodies. In practice however, antigen-matched VLP vaccines are likely to be applied in combination with live vaccines in the field, for maximum protection.

Transient plant-based expression is a cost-effective and scalable production platform. Based on the yields obtained here, conservatively estimated, one kilogram of infiltrated leaf material would be sufficient for the prime-boost vaccination of 10,000 chickens (1024 HA units (10 log_2_) per dose). In the field, however, the ND VLP vaccine would likely be applied as a booster to a live vectored vaccine given in the hatchery, which would mean that up to 20,000 chickens could receive a booster from one kilogram of infiltrated leaf material. A high antigenic dose of 1024 HAU was used in the present immunogenicity study to ensure measurable antibody titres were elicited following vaccination with the ND VLPs, but the prime-boost vaccination of insect cell-produced ND VLPs with an HA titre of 256 HAU (8 log_2_) was previously efficacious in chickens against viral challenge, resulting in an increased protection period, a reduction in viral load, and a shortened shedding period in comparison to a commercial live La Sota ND vaccine (Xu et al., 2019). At the latter dose, the potential number of chickens vaccinated per kilogram of infiltrated leaf material could increase up to 40,000. Furthermore, according to Ruiz et al. (2018), minimally processed VLPs could potentially be used as immunogen and would especially be beneficial for veterinary vaccines due to the cost implication of the reduction in downstream processes. Plant-produced F/HN VLPs that were only subjected to clarification yielded an HA titre of 1:512 (9 log_2_) i.e., double the effective ND VLP antigenic dose reported by Xu et al. (2019), therefore, a future aim is to investigate the toxicity an immunogenicity of minimally processed plant-produced VLPs in chickens. There is also potential for mass application of VLP vaccines *via in ovo* (Schädler et al., 2019) and intranasal administration (Earnest, 2009) with suitable adjuvants, which would be an enormous advantage for commercial poultry production.

Some of the biggest advantages of transient plant-based expression are undoubtedly its adaptability and short production time (Morris et al., 2017; Sainsbury, 2020; Tusé et al., 2020), which facilitates the production of antigen-matched vaccines. Infectious viral avian diseases often involve rapidly evolving viruses or there is a great diversity in viruses isolated from different avian species. The capacity to tailor a vaccine for a specific geographical region and or species is a major advantage of transient plant-based expression, as an antigen-matched vaccine confers the most effective protection against clinical signs of disease and reduction in viral shedding. In South Africa, the global leader in ostrich production with at least 75% of the market share (Department of Agriculture, Land Reform and Rural Development (DALRRD), 2020), vaccination of all ostriches against ND is compulsory for export purposes. The registered egg-produced ND vaccine (Struvac^®^ ND Plus, Deltamune, South Africa) for ostriches is formulated with a proprietary oil adjuvant to enhance its immunogenicity, but the local adjuvant-associated adverse reactions [a well-documented occurrence (Petrovsky, 2015)] devaluate the meat or hide. In addition to the potential application of a plant-produced ND VLP for the local ostrich industry, there is also a need for pigeon-specific vaccine against NDV variants. NDV strains that infect pigeons are referred to as pigeon paramyxoviruses (PPMV) and due to the important role that pigeons may play in spreading the disease as a result of prolonged viral shedding without clinical signs, strict regulatory measures (including compulsory vaccination) are imposed for racing pigeons (Aviomed, 2019; Miller and Koch, 2020). Using transient plant-based expression, antigen-matched ND vaccines can be developed for different geographical areas and/or for individual avian species (i.e., chickens, ostriches, and racing pigeons, respectively), which would provide maximum protection against these genetic sub-lineages. Vaccination of poultry and other avian species against infectious diseases like ND is of the utmost importance to protect export markets but more importantly ensure food security in many countries where poultry provides the primary source of dietary protein.

## Data availability statement

The datasets presented in this study can be found in online repositories. The names of the repository/repositories and accession number(s) can be found in the article/**Supplementary Material**.

## Ethics statement

The animal study was reviewed and approved by Ethics Committees of the University of Pretoria (UP); the UP Animal Ethics Committee, the CSIR Research ethics committee; and the Department of Agriculture, Land Reform, and Rural Development (section 20).

## Author contributions

CA: conceptualization. TS, CA, MO’K, NL: methodology. TS, CR, CA: investigation. CA, MO’K, NL: resources. TS: analysis, writing- original draft preparation. All authors contributed to the article and approved the submitted version.

## Supplementary material

Supplementary Figure 1 Phylogenetic tree of NDV isolates utilised for testing at APHA-weybridge.
