# Supplementary figures and images for: The production of Newcastle disease virus-like particles in Nicotiana benthamiana as potential vaccines
Source: Front Plant Sci. 2023 Feb 16;14:1130910. doi: 10.3389/fpls.2023.1130910 (PMC9978804; doi:10.3389/fpls.2023.1130910)

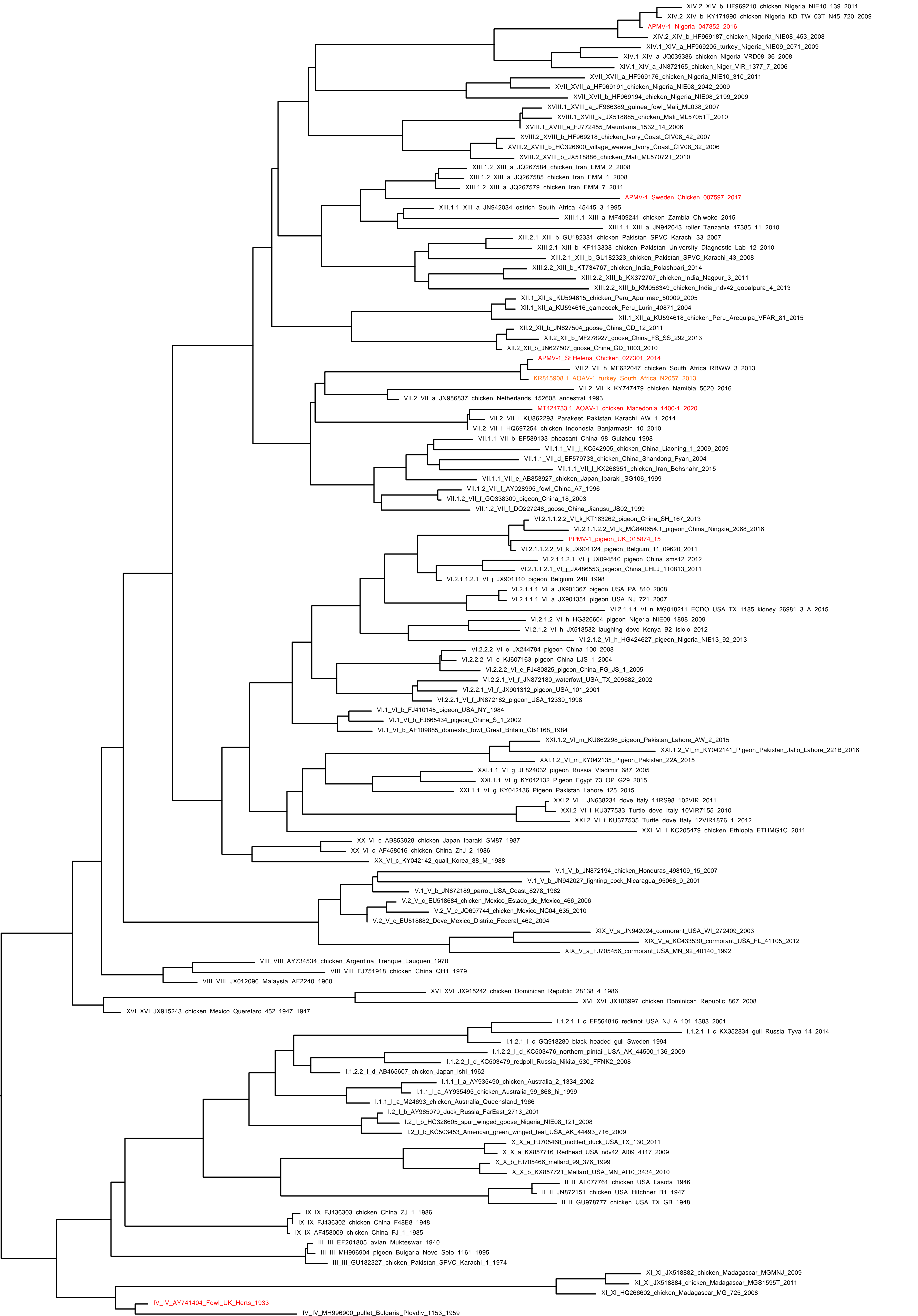

Supplement: Supplementary Figure 1 — Phylogenetic tree of NDV isolates utilised for testing at APHA-weybridge. [file DataSheet_1.pdf]
