# Supplementary material for: The production of Newcastle disease virus-like particles in Nicotiana benthamiana as potential vaccines
Source: Front Plant Sci. 2023 Feb 16;14:1130910. doi: 10.3389/fpls.2023.1130910 (PMC9978804; doi:10.3389/fpls.2023.1130910)
Supplement: Supplementary file 2 [file DataSheet_2.pdf]

A. NDV Fusion (F) protein: 81.4% coverage (95% confidence) and 48 unique peptides

LDGRPLAAAGIVVTGDKAVNIYTSSQTGSIIVKLLPNMPKDKEACARAPLEAYNRTLTTLLTPLGDSIRKIQGSV
STSGGRRRKR**FIGAIIGSVALGVATAAQITAAAALIQANQNAANILRLKESIAATNEAVHEVTNGLSQLSVAVGK**
MQQFVNDQFNNTAR**ELDCIKITQQVGVELNLYLTELTTVFGPQITSPALTQLTIQALYNLAGGNMDYLLTKLGVG**
NNQLSSLIGSGLITGYPILYDSQTQLLGIQVNLPSVGNLNNMRATYLETLSVSTTKGFASALVPKVVTQVGSVIE
ELDTSYCIESDLDLYCTRIVTFPMSPGIYSCLSGNTSACMYSK**TEGALTTPYMTLKGSVIANCKITTCRCADPPG**
IISQNYGEAVSLIDRRSCNVLSLDGITLRLSGEFDATYQKNISILDSQVIVTGNLDISTELGNVNNSISNALDK**L**
AESNNKLDKVNVRLTSTSALITYIVLTVISLVFGALSLILACYLMYKQKAQQK**TLLWLGNNTLDQMR**ATTRA

B. NDV Hemagglutinin-neuraminidase (HN) protein: 72.7% coverage (95% confidence) and 51 unique peptides

MDRVVNR**VVLENER**REAKNTWRLVFRITVLLLMVMILAISAAALAHSMGACTPR**DLAGISTGISKTEDRVTSLLS**
SSQDVIDRIYKQVALESPLALLNTESIIMNAITSLSYQINGAANSSGCGAPVHDPDYIGGIGK**ELIVDDTSDVTS**
FYPSAYQEHLNFIPAPTTGSGCTRIPSFDMSTTHYCYTHNVILSGCRDHSHSHQYLALGVLRTSATGR**IFFSTLR**
SINLDDTQNRKSCSVSATPLGCDMLCSKVTETEEEDYRSVTPTSMVHGRLGFDGQYHEKDLDTTVLFKDWVANYP
GVGGGSFINDRVWFPVYGGLKPNSPSDTAQEGKYVIYKRYNDTCPDEQDYQIRMAKSSYKPQRFGGKR**VQQAILS**
IEVSTSLGKDPVLTIPPNTITLMGAEGRILTVGTSHFLYQRGSSYFSPALLYPMIVHNKTATLHNPYTFNAFTRP
GSVPCQASARCPNSCITGVYTDPYPLIFHRNHTLR**GVFGTMLDDGQARLNPTSAVFDNISR**SRVTR**VSSSSTKAA**
YTTSTCFKVVKTNK**TYCLSIAEISNTLFGEFRIVPLLIEILKDDRV**

C. NDV Matrix (M): 21.4% coverage (95% confidence) and 9 unique peptides

MDSSRTIGLYFDSALPSSSLLAFPIVLQDTGDGKK**QITPQYR**IQR**LDSWTDSK**EDSVFITTYGFIFQIGNEEVTV
GVINNNPRHELLSSAMLCLGSVPNDRDLVELAR**ACLTMVVTCKK**SATNTERIVFSVVQAPQVLQSCMVVANR**YSS**
VNAVKHVK**APEK****IPGSGTLEYKVNFVSLTVVPR**RDVYRIPTAVLKVSGSSLYNLALNVTIDVDVDPKSPLVKSLS
RSDSGYYANLFLHIGLMSTVDKKGKKVTFDKIEEKIRRLNLSVGLSDVLGPSVLVKARGVRTKLLAPFFSSSGTA
CYPIANASPQVAKILWSQTAHLRSVK**VIIQAGTQRAVAVTADHEVTSTK**IEKKHTIAKYNPFKK

Supplementary Figure 1: LC-MS/MS-based peptide sequencing analysis for SDS-PAGE bands of approximately 56 kDa (A), 62 kDa (B) and 40 kDa (C), mapped against the Fusion (F), Hemagglutinin-neuraminidase (HN) and matrix (M) protein sequences, respectively, of Newcastle disease virus (NDV) turkey/South Africa/N2057/2013. Peptides identified with >95% confidence are highlighted in green, with 50-95% confidence are highlighted in yellow, and with <50% confidence are highlighted in red. Peptides were not identified for the non-highlighted regions (grey) of the respective sequences.
